# Reduction of a Dislocation Forwards of the Inferior Surface of the Fifth Cervical Vertebra

**Published:** 1849-01

**Authors:** 


*Reduction of a Dislocation forwards of the Inferior Surface of the Fifth Cervical Vertebra.* By M. Vrignouneau.—The patient fell from a tree, on his head, and lost consciousness, which, however, returned in half an hour; he then complained of violent pain at the vertex and back of the neck; the author diagnosed—how, he does not say—a dislocation forwards of the inferior surface of the fifth cervical vertebra. He bled the man, and ordered absolute rest, but without avail; and forty hours subsequently—speech having become difficult, the face injected, the respiration stertorous, and the pulse almost imperceptible—he determined to give him the chance of an attempt at reduction. For this purpose the man was seated, two assistants pressing firmly, one on each shoulder, while M. V. gently extended the neck. Partial extension rendered the speech stronger, and respiration freer, and emboldened the operator to proceed further. When he thought the extension sufficient, he carried the head and superior part of the neck backwards; this manipulation was followed by a snap, and from that moment the man recovered as by enchantment.—*Monthly Journal, from Journal de Connaiss. Medico-Chir.*

